# Facing disabilities in children with prenatal alcohol exposure: parenthood and stressors according to families and professionals' narratives

**DOI:** 10.3389/fsoc.2025.1671701

**Published:** 2025-11-27

**Authors:** Laurence Simmat-Durand, Stéphanie Toutain

**Affiliations:** Université Paris Cité, CERMES3, Paris, France

**Keywords:** prenatal exposure to alcohol, parenting style, parenthood (transition to), foster families, adoptive families, birth families, stressors, adverse childhood experience

## Abstract

**Background:**

Prenatal alcohol exposure can cause neurodevelopmental disorders affecting children's brain function, educational and social skills throughout their lives. International literature highlights the challenge for families of raising an affected child. Beyond their exposure to alcohol, these children face other negative events such as separation from their biological families, the mental health problems of their parents, and so forth, which will have repercussions on their childhood and their future health.

**Methods:**

This research used semi-structured interviews with 107 participants, namely 53 social and health-care professionals and 54 families (foster, adoptive or biological) of 62 children prenatally exposed to alcohol. All were recruited in two regions and a national association of parents in France. Families were diverse in terms of members, regions, ages, and social and cultural backgrounds. A thematic analysis was performed to distinguish parenthood styles and stressors.

**Results:**

The analysis allowed identification of three types of parenthood used to cope with these neuro-disabilities, independent of the legal status of the family: first, intensive parenting with a major involvement of the adults in charge; second, inclusive parenting seeking to normalize the child within the siblings, school group or society; and third, distanced parenthood where the disability was down-played and the child considered to have lesser abilities. In all cases, the stress, and the difficulties encountered, have a negative influence on the mental health of the parents and/or on the integrity of the family unit, with different modalities of shared parenting.

**Conclusion:**

Becoming the parent of a child with disabilities, even more so when the origin is attributed to stigmatized and stigmatizing behavior for both mother and child, is a long process that requires adequate diagnosis and management to avoid loss of opportunity. Parental investment, which determines the family trajectories, is based on social and cultural resources.

## 1 Introduction

Prenatal alcohol exposure (PAE) can cause neurodevelopmental disorders that affect children's brain function, educational and social skills throughout their lives. Fetal alcohol syndrome (FAS) caused by PAE is the most severe form of fetal alcohol spectrum disorder (FASD). FASD is an umbrella term that includes a range of conditions caused by PAE. International literature highlights the challenge for families of raising an affected child, especially because the diagnosis is somewhat difficult and often delayed (Oei, 2020). Children experiencing FASD are often removed from their birth family and placed in foster care (Simmat-Durand, 2005; Sennsfelder et al., 2024). Notably, French adoptive families faced this handicap for children in international adoption (now stopped).

FASD has a profound impact on brain plasticity—that is, its capacity to adapt. PAE can lead to cognitive deficits, behavioral disorders and learning difficulties. Secondary appearance of behavioral disorders and social adaptation is attributed to the nature of deficits and the frequently fragile life context (Jacobson et al., 2004; Streissguth et al., 1996). Early identification and care, as well as a stable family environment, can prevent over-disability (Garzon et al., 2022). The care of these families can be influenced by the representations that professionals have about alcohol misuse (Koski-Jännes et al., 2016).

Adverse childhood events (ACE) are factors that strongly influence life in adulthood, particularly health, but also socio-professional integration, family life, etc. The influence of different factors such as the use of psychoactive substances, depression or suicide attempts, has been highlighted (Felitti et al., 1998). Consistent results show that subjects who grew up in families affected by substance use have a higher risk of becoming dependent on alcohol (Dube et al., 2006) or other drugs, both due to their exposure and their environment (Douglas et al., 2010) and/or neuro developmental factors related to PAE. ACE are described as being likely to result in substance use, degraded health or an unfavorable socio-economic environment (Zlotnick et al., 2004). Furthermore, the precocity of these negative events could be an aggravating factor, reinforcing negative factors during fetal life, also referred to as developmental origins of health, or even epigenetics (Charles and Junien, 2012).

A quarter of women in France today consume alcohol during pregnancy (Garzon et al., 2022). FAS has a prevalence between 0.3 and 0.5 per thousand live births. The incidence of FASD is 10 times higher, and is the most prevalent cause of impaired neurodevelopment (Germanaud and Toutain, 2017). The world prevalence of alcohol consumption during pregnancy is almost 10%, and the prevalence of FAS was 15 per 10,000 people in 2012, with wide cultural variations especially within the European Union (Popova et al., 2017).

The parenting competence of birth parents is often challenged in the context of alcohol drinking (Olson et al., 2009), even when the parents are not abusing alcohol but rather wanting to share adult moments. The norm should be that becoming parents results in a decrease in alcohol consumption (Cook et al., 2022). In the case of drug or alcohol abuse, the capacity to be a “good enough parent” (Hays, 1996)—that is, respecting norms and not endangering the children—is questioned, notably in the evaluations made by child protection services. As previously shown, drug addicted women who become mothers fall at the crossroads of four sets of norms: criminal norms because drug use is illegal, social norms which set out what a good mother should be, the medical standards governing the follow-up of at-risk pregnancies and finally gender norms, because drug addiction is viewed as a massively masculine phenomenon. This conflict of norms leads to an overlapping of the sanctions applied to them, including the loss of custody of their children (Simmat-Durand, 2007). Mothers are expected to provide a safe environment and to act in a moral way, and this tends to exclude some categories of parents, including mothers who use drugs or alcohol or are in prison (Valentine et al., 2019).

The parenthood considered here is part of a very broad vision of kinship, described by Weber (2005), (2006) because it applies to: first, the lineage—that is, the biological mother or father, grandparents invested by the judge as trusted third parties; second, the kin group when uncles or aunts agree to raise the children but also in the case of adoptive families or in the case of the spouse of the biological parent; and third, the household in the case of foster families or temporary adoptions. Beyond blood and in-law configurations, we chose to collect the experience of the one who calls themselves the parent of the children at the moment we met them. The relationship is a practical one in the sense of the parent who raises the child. It has been developed as shared parenthood for long-term foster care, where the children navigate between two families (Cannaert and De Wilde, 2025). Therefore, we chose to consider all living arrangements and to use network-oriented family definition (Schneider and Kreyenfeld, 2021).

Parenting styles are usually described as the “emotional climate” in which parents' attitudes are conveyed; they are assessed with typically developing children to allow comparison with children with disabilities, but are less commonly assessed among children with developmental disabilities (Marston et al., 2022). Parenting styles are commonly categorized as authoritative, authoritarian or permissive, according to Baumrind's typology. These are based on parenting behaviors and a psychological approach, and Maccoby and Martin (1983) later added non-involved or negligent parenting as a fourth style.

As illustrated through autism, which is another neuro-developmental disorder, the notion of parental responsibility in the context of a child's disability has been greatly transformed, resulting in a shift from disqualification to a partnership with health teams and a request for expertise from the child's parents (Borelle, 2017). This analysis nevertheless finds its limits in the case of FASD, where parental responsibility is direct, at least regarding the biological parents (Zabotka et al., 2017). Unlike autism, FASD has received little attention and dedicated associations receive less consideration on the political scene, especially as the behavior of the mother is questioned in the pathology and alcohol is a sensitive issue in France (Dumas et al., 2018). In France, parent associations are primarily established by adoptive families or professionals who frequently associate with biological families (Lamblin, 2010; Metelski et al., 2016). The experiences of adults who have parented or are parenting impacted children remain under-researched, particularly for biological families (Rennie et al., 2024).

An important part of the literature on parents of children impacted and living with FASD has focused on identifying their needs, in training and daily life, to take care of these children. Kautz-Turnbull et al. (2023) included 46 caregivers having a child aged 3–12 with confirmed FASD diagnosis or PAE in a programme to examine their parenting-related stress. Pruner et al. (2020) compared the experiences and needs of biological and adoptive parents confronted with FASD in their children. Weinmann et al. (2021) carried out research in Germany through a quantitative survey among caregivers and professionals. They concluded that the needs of caregivers were largely unmet and the help they received tends to be insufficient. Phillips et al. (2022) examined the impact of FASD on families. They highlighted the effects on caregivers, families and unaffected children, concluding that there is a lack of family-centered approaches to address the needs of this population.

Research about parents' daily difficulties and how they manage the needs of these children remains limited—with little research on the construction of parenting around children with FASD. Michaud and Temple (2013) collected the experiences of five women: they described raising their children in ways that did not align with conventional parenting techniques, and they experienced guilt for not being able to meet their complex needs. Positive interactions between the caregiver and the child contribute to optimal development (Reid and Moritz, 2019), while non-optimal environments turn small vulnerabilities into larger deficits (Finnegan, 2013).

A second part of the research on families with FASD children focuses on their mental health, that of the children or that of the parents. A study on 24 foster and adoptive families was conducted with a perspective of developmental psychopathology (Petrenko et al., 2019). They explored the protective actions undertaken to protect children from secondary adverse events and the stress these actions developed. Eight adoptive families of 16 children with FASD were interviewed in Canada (Balcaen et al., 2021): participants were women married to men, all more than high school education, six with good incomes. They revealed difficult management of individuals with FASD, family cohesion endangered as well as psychological health, experiences of a crisis such as substance use, rapes, attempted suicide, all in one safety issues. Harding et al. (2022) focus on caregivers ‘experiences of suicidality among children and youth with FASD, through six interviews with five caregivers (4 adoptive and 1 trustee). They distinguished individual factors such as traumatic background, siblings with FASD, and community level factors such as delay in seeing a psychiatrist. Langevin (2023) in Canada related the experience of 10 adoptive or foster families about their experience of rising children with FASD in the context of Child Welfare protection. The analysis is mainly based on children's behavior in different contexts. Paley and al. suggested that in families with low economic status, having fewer family resources to support their children was a predictor of higher maternal stress among biological mothers (Paley and O'Connor, 2009).

To examine the way in which parenthood is built around children with FASD and the stressors that this induces, we have constructed a research device questioning the widest possible variety of professionals and families confronted with this condition. The interviews were about the daily functioning of families, to analyse their parentings choices, ways to cope with the complex needs of exposed children, the consequences in terms of stress, of mental health or family cohesion. The parenting styles of these families were then grouped into three main modalities following a thematic analysis. As an aid to comprehension, some specifics about the French organization for disabled children are mentioned in Appendix 1.

## 2 Materials and methods

This research used in-depth semi-structured interviews with 107 participants: 53 health care and social professionals and 54 families (16 adoptive, 13 foster, 25 biological) of 62 children suspected of having FASD or PAE. All were recruited in two regions through care or diagnostic facilities, as well as a national parents/patients support group, in France, to ensure heterogeneity of their profiles. Data were collected from 2023 to 2025. Families were diverse in terms of membership (39 mothers, 9 fathers, 2 grand-mothers, 4 couples together), regions, ages, social and cultural backgrounds. The data also included exploratory online questionnaires completed by 35 families (33 adoptive, 2 biological), with open questions describing living conditions with the children.

After identifying the structures involved in diagnosing or taking care of children in the two regions, we met with professionals so that they could introduce us to the families. The parents were interviewed either in these places (8 distinct structures), in a room made available to us preserving confidentiality, or at home in two cases. The support group gave us a list of families they contacted before and the interviews were made online.

Professionals who participated were from various structures, including health, education, child welfare or justice organizations. They were interviewed about their knowledge of the effects of alcohol on children, their education or experience in this field, as well as their practices. They were also asked to give at least one example of support/care in which they had participated. Families were asked about their family structure, the life events they felt worth revealing, the difficulties with the children, their care path and whether there was a diagnosis. They were also asked to detail school and care trajectories, the joys and difficulties of daily life, as well as the positive and negative aspects of life with the children.

The duration of interviews ranged from 39 min to more than 3 h, mainly depending on the conditions of the meeting (mean duration 81 min). The shortest interviews took place in hospitals, as we had to respect the constraints of the consultation schedules. For this reason, interviews were often 59 min exactly with the professionals. All families and professionals received detailed information and gave their written informed consent to participate in accordance with the terms authorized by the Paris Cité University's ethics committee (CER no. 2023-124). No incentive was provided for participants but the researchers will share the study results with the support groups and medical services. To preserve anonymity, the ages were grouped into large groups (< 40; 40–59; 60+) and the locations not identified.

All interviews were conducted by the two researchers, together or separately. This type of interview on sensitive topics and vulnerable populations has been conducted for more than 20 years by the first author, which has been a great asset in contacts with professionals and adoptive families. The choice not to call on intermediaries, translators or staff involved meeting only people with a minimum mastery of French. Some statements are difficult to transcribe exactly. Our position as senior lecturers, white, native French -speakers, was able to put some interviewees in uncomfortable positions, compensated by our availability and great empathy, being both mothers, one of a child with neurodevelopmental disorder and the other with the experience of an adult member of her family with FAS, and our extended knowledge of the consequences of such disabilities. Some interviewees gave the impression of testing our knowledge on the subject before delivering more specific elements of their experiences, notably the professionals and families involved in the associations. For most of them, the possibility of being listened to outside of care or administration has most often been expressed as appreciated. The discourse held was nevertheless influenced by our position as a researcher or the place of the meeting.

All interviews were transcribed verbatim and anonymized. All data were coded in NVivo14 software by both researchers. From the data of the interviews, codes were created, which were then grouped into sub-themes and themes to create an analysis structure. The themes ranged widely and only a few aspects were used for this article, where a thematic analysis was performed to distinguish parenthood trajectories or styles. The idea was not to test the parental styles defined in the literature but to create a typology derived from the data, using a grounded typology (Charmaz, 2006). The quotations illustrating this article have been transcribed in French as they were spoken, with their possible instances of misspeaking or local expressions. These idiosyncrasies have been carried over into the translations.

## 3 Results

The families interviewed were very diverse in terms of their legal status: biological, adoptive, trustees or foster care by judicial decision. Foster families in France are paid, and those we met provided long-term foster care and identified themselves as parents. The age of the parents ranged from 32 to 68 years. The efforts made to meet fathers allowed the sample to include six fathers, and one couple, out of the 25 biological families. The foster carers were almost exclusively women, except for two, just like adoptive families where two men participated at the same time as their spouses and one alone. In terms of siblings, there was an average of 3 children in total (range 1–9). Most families had a child with FASD, but nine families had two such children and one raised three. The age of the children varied from 1 to 27 years, with an average age of 15 for the first child, 10 for the second and 4 for the third. The families came from all social backgrounds, from senior executives to unemployed women with a very low educational level. Some of the biological and foster families were of minority ethnic origin. The professionals ranged in age from 22 to 75 years and were mostly women (38 out of 53) with an average age of 48 years. Table 1 shows the characteristics of the interviewees whose narratives are quoted in this text.

**Table 1a:** Profiles of families quoted in this text.

**Number**	**Description**	**Gender**	**Age (years)**	**No PAE/PAE children**	Age child1^*^	Age child2^*^	**Duration (mn)**
1	Foster mother	F	≥60	3+1	27		55
2	Foster mother	F	< 40	3+1	9		68
5	Adoptive mother	F	≥60	1	17		136
6	Adoptive mother	F	40–59	1+1	19		131
7	Birth father	M	≥60	2+1	17		61
15	Paternal grandmother	F	≥60	1	9		65
19	Birth father	M	≥60	5+1	6		74
33	Foster mother	F	40–59	3+2	15	10	63
36	Foster mother	F	40–59	2+2	18	6	80
38	Birth mother	F	≥60	2+1	24		129
44	Adoptive father	H	40-59	0+2	20	19	77
45	Birth father	M	40–59	1	9		67
49	Adoptive mother	F	40–59	2+1	12		131
51	Birth father	H	≥60	3+1	25		101
61	Adoptive mother	F	≥60	1	19		127
64	Adoptive mother	F	≥60	2+1	25		84
65	Adoptive mother	F	≥60	1	19		70
66	Foster and adoptive mother	F	40–59	4+1	11		80
67	Foster mother	F	≥60	2+1	6		95
71	Adoptive mother	F	40–59	1	19		81
72	Adoptive mother	F	≥60	1	25		108
73	Adoptive mother	F	≥60	2	20	20	142
76	Trustee parents	F+M	< 40	2+1	8		64
78	Foster and adoptive mother	F	≥60	6+1	13		39
Q9	Adoptive mother	F	40–59	3+1	22		
Q18	Birth mother	F	40–59	2+1	22		

^*^Impacted child.

**Table 1b:** Profiles of professionals quoted in this text.

**Number**	**Description**	**Gender**	**Age (years)**	**Duration (mn)**
14	General practitioner	M	40–59	71
24	Neuropsychologist	F	40–59	71
29	Coordination nurse	F	40–59	57
56	Specialized educator	F	40–59	132
75	Network director	M	40–59	150
83	Social worker	M	40–59	72
87	Neuropsychologist	F	< 40	69

The analysis of the interviews allowed us to identify three main types of parenthood to cope with FASD, independent of the status of the family. The main consequences in terms of mental health and families‘ problems are related for each type.

- *Intensive parenting*, which involves a major involvement of the adults in charge, often an over investment, but also more often a feeling of burnout or exhaustion among the parental caregivers.
- *Inclusive parenting*, which seeks to normalize the child among the siblings, school group or society. These parents experienced stigmatization and discrimination which generate stress.
- *Distanced parenthood*, where the children's disability was downplayed and their abilities viewed as inherently limited or a strategy to manage respite in the long-term care of the children.

### 3.1 Intensive or protective parenting

In the international literature, intensive parenting is described as the most widespread form in contemporary society, associated with an idealized vision of the mother, building the perfect baby.

The discovery of the child's difficulties was often a shock, because the families were totally ignorant about the child's health, although some families may have been informed of probable problems, or in the case of adoptive families, the file mentioned “specific needs”. For instance, this adoptive mother explained that another cause was imputed for a long time for explaining her child's difficulties:

*No, no. It was delay, psychomotor delay*… *Delays were still mentioned. At first, of course, I blamed it on the fact that for 21 months he had not been taken care of. Since*… *During the first months, he made a lot of progress very quickly, including*… *Ah yes, because he did not walk when we had him. He walked three months after we got him*. (Adoptive mother, ≥60 years old, one child with PAE, n°65)

Parents undertook actions to ensure the children's protection, mitigate the effects of the pathology and avoid secondary problems. These actions required a major involvement of the adults in charge, which sometimes led to an over-investment to fight adverse events. In this category, most mothers gave up work or were working part-time or at home. Others provided significant financial or family support for childcare. A significant proportion participated in an association or collective to inform, help and support the parents of children with FASD.

All narratives from parents in this type of families reported the heavy load, both organizational and in terms of well-being, even mental health. These actions focused on the remediation of disorders often leading to a destruction of the family unit, by divorce or cessation of foster care, the termination of the mother's professional activity and an overinvestment that seems endless to them, generating concerns for their child's future.

#### 3.1.1 Actions for care/cure

The first area in which this action was carried out was to remedy the health condition of the child. The families multiply the steps and consultations to obtain a diagnosis and set up multiple follow-ups.

The care of these children was often expensive and time consuming, due to multiple appointments with different practitioners (e.g., speech therapist, occupational therapist, psychometrician) especially when parents did not depend on a CAMSP but on the private practice. The assistance received by parents also depended on their ability to obtain recognition as having a disabled child, which opened up financial or school–life support.

*You must run everywhere and then again, the general council, he can ask for you to have appointments at the same time, so it's up to you to fight to say that if I'm there, I can't be there, so it's constant fighting whether in school or with the general council or for appointments, we fight all the time*. (Foster mother, < 40 years old, two biological children, two fostered children with PAE, n°36)

All possible remedies, such as homeopathy, equine therapy, reflexology and so forth, were used, including alternative medicine. In the absence of a diagnosis, all resources were mobilized, often with very high costs, by a number of stakeholders from the private sector or outside the sector. The examples cited were quite diverse, including magnetizers, kinesiologists, homeopathy, Bach flowers, Feuerstein method, naturopath and art-therapy.

*We're not ruling out anything that might help him. I mean, he was fine. He wasn't*…*. Well, it was still super complicated. We did some remediation of the infant's primitive reflexes. Well, speech therapy went on. Anyway, speech therapy was done until the age of 15, almost. [*…*] We did another thing too. I had forgotten about remediation, spelling, etc. And he has always been well trusting, because we never forced him, but we had everything we could offer to help him move forward*. (Adoptive mother, ≥60 years old, two adopted children of whom one with PAE, n°6)

Many stories showed this accumulation of complex care pathways, in search of the management that would have made it possible to counter the fate of the child's disability and bring him back to a standard from which he was escaping. The second major axis of this action concerned the schooling of the children. For many of these families, the loss induced by their neurodevelopmental difficulties led to forgoing brilliant studies, especially for those belonging to favored socio-professional backgrounds.

#### 3.1.2 Schooling

All possible solutions were also explored for schooling, including private schools, alternatives and even home school. Some modalities were made possible with the mother having no outside activity or stopping working.

*And from the moment he was home, he didn't have any health problems, there was no pressure. I said, no, you don't come back, it's over. And then it was good. That is to say, I was able to completely take over the entire sixth programme; reworking with him the techniques he had never been able to acquire*. (Adoptive mother, 40–59 years old, two adopted children of whom one with PAE, n°6)

Schooling was often described as a long-term struggle to follow a course of study until adulthood, despite disability-related orientations.

This was particularly true when school supports were not obtained or in place. Inclusive education policy in France is encountering extremely long delays for the review of files by the administrations in charge, and the chronic lack of caregivers in class (AESH), as explained by professionals as well as families:

*The AESH for the little one, I fought for her, and here it is, but there is still no AESH, so she is in pain, and then she completely gave up, even if at the beginning when she arrived she was hyper brave, willing, but now she is no longer, so at the school level we lost her, so for me it's a pain because she is conscious*. (Foster mother, 40–59 years old, two biological children, two fostered children with PAE, n°36)

Parents spent a significant amount of money on expensive tests, including abroad, to search for the best school orientation, with the risk of no longer meeting the criteria to benefit from medical or social aid.

*So, she had a speech therapist. Then, she had a CMPP in town. So, there was the paediatrician. There was a reference teacher. I had her do so many tests – so, psychometric, logical-mathematical test, because she had difficulties, but also strengths. And the paediatrician told me I will have to overemphasizebecause otherwise she will not be entitled to an AESH*! (Adoptive mother, 40–59 years old, one child, n°71).

The paradox of these actions to remedy the difficulties of the child is indeed that they lead to depriving the child of any public help, related to a percentage of disability. But since some assistance is linked to the parents' income, a part of the families encountered were not entitled to it anyway and relied only on their own resources.

Parents who were highly invested in their parental role also tended to seek to optimally develop their child's abilities, including through sports activities or arts. For them, this meant introducing as many extracurricular activities as possible to their children to develop numerous skills, they consider also therapeutic, in terms of coordination for instance.

#### 3.1.3 Involvement and advocacy

By looking for resources from all sides, some families develop skills in the field of FASD disability, which literally became their “first profession”. This included adoptive mothers involved in associations, but also biological mothers wanting to testify in all media, or foster mothers trying to make things change.

*And, well, at the end of my testimony, for example, she was always there to support me. Because it's not obvious. How do people tell me that? It's not easy to get naked in front of people. And I've been doing this for 12 years [*…*] well, I was there all the time. How to say? How was I? I don't know. But I was still there*. (Biological mother, ≥60 years old, three children of whom one with PAE, n°38).

*And I assure you, it was a real fight. This is the fight of my life, let's say. That, for me, really, I talk about it around me. [*…*] I talked to the psychiatrist. [*…*] That's really who heard me. And it's good to be heard, too, to come up with information. I'm on my way*… *You know, even at the very beginning, I had not seen the psychiatrist, but I had seen the educator who questioned diagnoses. I was coming in with documents from the association. And I put them on his desk. Maybe I had to say that. Now, I'm a bit of an activist in this*. (Foster mother, ≥60 years old, three fostered children of whom one with PAE, n°67)

In such cases, mothers did not hesitate to take training to accompany their child along the chosen path, or to become their child's employer and gave them every opportunity to flourish or even to serve as an example to other families, notably through advocacy actions.

*This is a bit of the spirit of our association, I am there for them, if by luck I manage to make sure that [Child] actually has a professional career and that the model, the accompaniment that I would have put in place for him is relevant as far as it serves to others*. (Adoptive mother, ≥60 years old, one child, n°5)

As often noted in the literature, this energy spent on organizing the different cares for children has consequences in terms of mental health and family cohesion, reported by the interviewed families.

#### 3.1.4 Mental health and familial consequences

This intensive parenting more often resulted in a feeling of burnout or exhaustion of parental caregivers, even more if the help provided was not adequate because the diagnosis had not been made. The topic of family burdens was recurring, making it challenging for families to find help due to the situation being misunderstood by most professionals. Anxiety and guilt were at their peak for mothers who confess to drinking during pregnancy and dedicated their lives to the children as a redemption. For some women, conversely, their mental health was evoked as the cause for alcohol use during pregnancy:

*Unfortunately, at my second pregnancy, instead of feeling better, I felt a very important anxiety [*…*] This anxiety led to the intake of alcohol that I did not talk about and that I thought without consequence. [*…*] I think that an improvement in the psychiatric management of depressions and anxio-depressive syndromes would avoid the experience I had. Even if I do not absolve myself of having made this monstrous mistake and that I bear the guilt on a daily basis. (Birth mother, 40-59 old, 3 children of whom 1 PAE, Q18)*

With the daily burden of caring for these children, the lack of respite cropped up in many stories, almost in all types of families. For instance, some foster families gave up taking vacations for many years:

*Yes, but imagine that when I started working, we had a Down syndrome in the beginning. Then I had Child. Well, the holidays, so as not to disturb the children, we did not leave, but we did not leave on holiday. So, I can tell you how long I've been on holiday since I started working: two weeks. Two weeks, I was able to go on holiday when my boy got married. I said no, now I'm taking a two-week break. So, I put Child, I asked to place Child at someone, a friend [in the end she took him with her]. (Foster mother, Age 60*+*, 4 children of whom 1 PAE, n*°*1)*

Several mothers used the expression that they felt they were going crazy, faced with the crisis of the child, their inability to find solutions, the lack of empathy of the caregivers or the absence of a diagnosis that did not allow words to be put on what they lived.

*At the hospital, the interns, when they told me, “we can't do anything for you”. [*…*] If you say that you can't do anything for my daughter, I'm going to go crazy. I'm going crazy or she's going to kill me. Because that's what's going to happen, if you can't do anything for her. Well, they told me, “get back in line at the emergency room. We welcome you as an emergency”. (Adoptive mother, Age 62, one child, n*°*61)*

Finally, the huge investment in the child's education led to successive renunciations of other aspects of life, such as marital, social, leisure, etc.

*And I'm feeling guilty that I didn't have enough time for Child. If I had, maybe I would have this. And I must say that I don't mean at the end of my life, but in the fall of my life, because I'm 62 years old, I had no life. I couldn't do it. (Adoptive mother, 60*+*, 1 with PAE, n*°*72)*

A final aspect that we cannot develop here is the break-up of several couples, adoptive families in particular were reduced to adoptive mothers, their spouse choosing to separate because they no longer had a place. The other children were often “sacrificed” and the resulting regrets in the mothers interviewed were very evident. Their worries grew with the ages of the children and the women who were close to sixty feared for their future.

If this intensive model seemed to be more often used by adoptive families, foster families could also provide intensive assistance to “save” the child. This might also be the case for a biologically related parent, such as a grandmother or aunt, who was entrusted by the judge. In biological families, the desire for reparation was often strong due to the guilt linked to the behavior that caused the FASD disability.

The second group of families, unlike the intensive family remediation efforts, tended to downplay FASD disabilities in all aspects of daily life by integrating children as much as possible into typical groups.

### 3.2 Inclusive parenting

These parents sought to normalize the children among the siblings, school group or society by considering them as special (e.g., an artist or dreamer), with different skills. It was challenging for them to face the demands of school and the perception that their children were late-developing students who required repetition or orientation. They therefore tried to continue in the conventional school system, regardless of results. They refused the diagnosis and special management because it would put a label on their difficulties. By default, this group also included families who did not have the financial means to look for alternatives. Some families just reject the diagnosis of FASD, as explained by this professional:

*Yes. With really this thing of*… “*I don't want my child to have that label”. I think… well. I talked about it at length, with the mother…. It was really not at all*… *Conceivable as a diagnosis. After, it is a family that is in the… Who also refuses a lot of other things, even in care, etc*. (Neuropsychologist, female, < 40 years old, n°87)

Contrary to what happened in intensive families, the children' skills, other than their academic evaluation, were more often highlighted as a main component of their character or attitude.

#### 3.2.1 Attitudes toward care

These children were cared for in an unspecific care pathway, in the public sector and outside of a family approach. The children's difficulties were believed to be caused by somatic pathologies, which were often dismissed as trivial. Here are two examples, showing that this way of taking in care children who are affected concerned the different types of families:

*There, she goes to school from Monday until noon on Friday. In the afternoon, she doesn't go. She goes to CMPP. The CMPP takes over in the afternoon. […] But right now, she doesn't need to see a psychologist*. (Paternal grandmother, ≥60 years old, one fostered child, n°15)

*At that time, the doctor told me, it's normal. These are normal disorders. She needs to adapt. She is completely normal. There are no health issues, nothing. She seemed physically healthy, in any case*. (Adoptive mother, ≥60 years old, three biological children, one adopted with PAE, n°4).

The pathways via the CAMSP were generally appreciated by the parents, even if places were limited and waiting periods often long. Therefore, the children stayed there for a short time since the reception is limited to 6 years old.

*They did what was necessary so that I could get the taxi. They were coming to take Child and me to come. And so, she went at the CAMSP. She had the psychomotor therapist. She had the speech therapist at the age of two and a half, three years I think. The psychometrician and the doctor*. (Paternal grand-mother, 60+, trustee one child with PAE, n°15).

*She was registered in the CAMSP. We had a place. Hallelujah. Child was at the CAMSP in Town with an exceptional psychomotor therapist. Very, very well. It lasted for two years because afterwards, I think there is an age limit. She has made huge progress. Child, she is adorable. It's almost, it's not a fairy tale but not far that is to say that even with her difficulties, she develops* (Adoptive father, 40-59, 2 adopted children with PAE, n°44).

The inclusion policies of several countries, including France, involve not grouping children according to their level of disability, but integrating them into regular schools, with or without an assistant, sometimes partly in specific classrooms.

#### 3.2.2 School standardization as a main objective

The biggest challenge for these families was school failure and the grieving of an academic journey, including in family environments with high levels of education. The families either refused a school orientation outside of ordinary schooling, or did not seek or refuse that their children benefit from school support, in order not to single them out, or designate them as disabled, as expressed by these three families, biological, adoptive and foster:

*Father: So there, in college, they offer me to orient him, at a time, on the family structure, I no longer know how to call*.

*Researcher: the MFRs [Rural Family Home]*

*Father: That's it, and then, how about that, “no, I said, no, no”, because [Child] ten years old, said “dad the sun will explode”, what he had read, he had looked, he is hyper sharp on domains. So, when he learns something, he goes all the way to the end of the thing, and then after he stores it. It's sponge, it's an unordered hard drive and he accumulates. It does not allow him to live in society, it is a hard disk*. (Biological father, ≥*60 years old*, four children of whom one with PAE, n°51)

*Mother: And so, he was in regular class*.

*Researcher: There was no AESH or CLIS or whatever?*

*Mother: No, because I did not, no, I was not asked. And I, let's be honest, I did not see my son as a disabled child. Maybe I hadn't accepted it yet*. (Adoptive mother, ≥*60 years old*, one child, n°72)

*Researcher: And in school, he had tried to, there was someone to help him in class, or not?*

*Mother: No, no, not at all, he didn't get anything*.

*Researcher: Okay. So, in fact, he never had a tutor?*

*Mother: He never had a school help, except, well, as I said to you, the girl, the person who took care of the library, to be with him at hours, at hours well, very precise, what*. (Foster mother, < 40 years old, 1 biological child, three fostered children of whom one with PAE, n°2)

Despite all these efforts to ensure normal schooling, difficulties remained also for the school system that was not adapted.

*Because he didn't do anything at school. To avoid permanent exclusion and allow teachers to “breathe”, we have multiplied the internships of 2 or 3 weeks, where difficulties began in the second week... It was necessary each time to find the internships, adapt the family organization accordingly, support our son in his new professional learning*.. (Birth mother aged 40–59, 4 children, one adopted with PAE, Q9)

The children's academic difficulties were underrated or not reported as worthy of interest. As a result, the emphasis was generally placed on their good character or exceptional gifts in other areas of their lives.

#### 3.2.3 The good children and their gifts

The children's emotional skills, for example, were highlighted to show that they are not comparable to a disabled child. The child's good mood or hugs in particular were interpreted as proof that the children were adapted to family life and provided gratification to those who care.

*Then, next to that, she is a very affectionate child, very loving. In terms of… In quotes, of mom's satisfaction, I am perhaps much more satisfied than many teenage moms, because they spend their time telling me that I am an extraordinary mother, that I am beautiful, that she loves me, that she loves how I am*. (Adoptive mother, ≥60 years old, one child, n°61).

*Because [Child] is always smiling. She doesn't show her disability, what. So…*. (Biological father, ≥60 years old, three children of whom one with PAE, n°7).

Some parents stressed their children had gifts, and playing music or drawing could sometimes help reveal these talents. For these parents, practicing sports in an ordinary environment was a way to integrate socially, to open up to others and broaden one's circle of acquaintances. It was also a vector of self-esteem for the child.

Inclusive sports practice often represented a kind of challenge for parents but also a way to feel that their children were like others. At times, the educator failed to recognize the hidden FASD disability as a problem in understanding rules and fragility. It seemed that no arrangement was possible, as it might be in a school environment.

Maintaining unspecialized medical or educational paths also has an immense emotional or mental health cost for parents who encounter different stigmas regarding their child or various forms of discrimination.

#### 3.2.4 Addressing stigma, discrimination and guilt

This inclusion in an ordinary environment, without recognizing or revealing the child's disability, was paradoxically described as a source of stigmatization or discrimination, the difficulties being then attributed to ‘poor education' and the parents blamed. To avoid facing discrimination against their children, these families tended to choose to modify their environment, with the risk of isolating the family unit.

*We sort a little at the level of family and friends because all those who have trouble with noisy environments, we see less and less. But that is part of our job. And we know that in this job, we tend to a little… I will put the same word again, sort it out. In people, there are some who can't stand it at all. There are some who show pity. And it is something that I can't stand. Because these children, they don't need pity*. (Foster mother, 40–59 years old, three biological children, one adopted with PAE, n°49).

*It is a handicap that is invisible. So, it's true that we had teachers who thought I was a mother, the mother annoying. Because my son, if he couldn't do it, maybe he wasn't working hard enough or trying hard enough or…*. (Adoptive mother, ≥60 years old, three adopted children of whom one with PAE, n°64).

These adverse experiences often lead to a withdrawal into the family unit, generating isolation, fatigue or burnout, with a background of responsibility feeling or guilt from the parent who cannot integrate the child.

*But how do we do that? It took three of us to control it when it exploded. It's a bit... He had a strength that was increased tenfold when he left for these crises. And I, in fact, couldn't control him. […] Me, I was completely out of stock. So, I had a burn-out. I had left my job. I couldn't anymore. (Adoptive mother, 40-59, 3 adopted children, 1 with PAE, n*°*49)*.

*It's complicated between my husband and me. The advantage is that we both have a desire to protect the couple. To protect Child. To protect our family. But my husband is more destitute than me regarding all this. Because I am getting help from a shrink. I have enriching social activities. Him too, a little bit. He has associative activities. But emotionally, he, he has a hard time. He is tense. He is tense, tense, tense*. (Adoptive mother, over 60 years, 1 adopted child with PAE, n°61).

Far removed from these first two categories of families, the third identified type, named distanced parenthood showed a greater distance to the difficulties of the children and the ways to address them. Resignation was sometimes shared by families as well as the professionals involved.

### 3.3 Distanced parenthood

A number of the professionals described families they believed were negligent in the care or education of children. It was obviously more difficult to gain direct testimony from the families on this disqualification. In contrast, some families believed that early childhood care in collective structures, such as nurseries, was particularly careless and unsuitable for children's development. The stories of parents, especially fathers, tended to show forms of resignation toward problems reported by the school. For these families, the FASD disability was downplayed and the child considered to have lesser abilities. In others, the families wanted to hide the child's difficulties, for different reasons. Character traits were not attributed to a medical condition but to poor management of the child.

*And then, there are the families who can have either a delegation of disability or a rejection of the child's disability and, here, it may take a little time, even long before they think there is a real problem that needs to be addressed. And so, this is also dependent on the degree of disability related to neuro-developmental disorder*. (General practitioner, male, 40–59 years old, n°14)

In this type of attitude, the difficulties of the children were not especially noted or were attributed to somatic or banal causes.

#### 3.3.1 Diagnosis and care

According to some professionals, foster families and some birth families were often quite distanced from the needs of the children because they had several in care and there weren't many solutions so that they could go to many appointments while caring for other children. This could have led to a long delay in diagnosis because the children's difficulties were underestimated, attributed to the deprived environment from which they come. This foster mother expressed that perhaps some foster families underestimated the disabilities to avoid medical appointments:

*Researcher. Well, yes. And she was, however, she had an AESH, all her schooling?*

*Mother: No, not at all. Now we start screening. I said, 13 years old is not normal. No, but I do not blame the other foster family, but not to go to the appointment everywhere, I think, she veiled to say that everything is fine*.

*Researcher. And the school didn't react either?*

*Mother: No*.

*Researcher: That means there are a lot of people who haven't reacted*.

*Mother: That's it. As long as the foster family doesn't say anything, the school won't move*. (Foster and adoptive mother, ≥60 years old, two biological children, four fostered of whom one with PAE, one adopted with PAE, n°78)

Some families believed in the power of medicine to find a solution, to fix what seemed to be temporary problems for the children. Such care was performed in accordance with the prescribed result without attribution of a definitive character to the disorder.

*I go to school, go to the speech therapist, go to the hospital, to the psychologist, all that sort of thing. I did not let go, because Dr X, he said sooner or later it will unlock and when it will unlock, he can do anything he wants! Well, now he is okay, with friends or girlfriends, they work, they earn pay…* (Biological father, ≥60 years old, six children of whom (at least) one PAE, n°19)

For children in a foster home, the long-term medical or paramedical follow-up could be hampered by placement disruptions in the child welfare structures, leading to breaks in care:

*He was followed by a psychometrician, but after that when he went to [City] it could no longer be done, so we stopped everything there, he had psychological follow-ups but it did not work because he did not speak*. (Foster family, 40–59 years old, two biological children, two fostered with PAE, n°36).

Care was often accepted only under pressure from school and specifically to buy peace at school.

*The year of the middle kindergarten section went wrong. So, that's when he was hospitalized. And that's the end of the school year where he took Ritalin. In fact, it calmed him down and we ended the year peacefully. Unless it made him completely drowsy. And, I didn't like to see him in that state. Well, the idea was not to shoot him. So, I did it to please the teacher, very honestly*. (Biological father, deceased mother, 40–59 years old, one child with PAE, n°45)

The stories notably of professionals showed these children ‘under the radars' for many years. This was all the more possible as recent education policies were to no longer make children in difficulty repeat, which allows them to cross all school levels, postponing the problems to the end of compulsory education on the 16th birthday.

#### 3.3.2 Poor schooling

Several situations were described where the children's needs were not identified, including because they were not in school, or were in school inconsistently. However, the school tended to be the first point of alert in assessing children's needs.

*Social Worker: No, I think the mother, her children, I think, for their schooling, they were more out of school than they were in class*.

*Researcher: But the college, he had to, the teachers…*

*Social Worker: The college, we say, it's not that they didn't want to have a team meeting. When they had meetings, the mother did not come to. She never came. [Child], I think he was more at home than at school*.

*(Child welfare social worker, male, 40–59 years old, n*°*83)*

In particular, there were cases in which the young people changed foster families. For these children, school follow-up was inconsistent and the professionals were late in realizing that the children had not actually been educated.

*But I also think, sometimes, of young people, in fact, for whom there are quite a lot of behavioural disorders, or scholar drop-outs, or things like that. So, change of host family. So, a foster family who would try to set up, I don't know, put on waiting list of young people, I don't know where. In the meantime, he has changed foster family. And it means that we have young people at 15 years old, in fact. Nothing has happened. (Neuropsychologist, diagnosis centre, female, 40–59 years old, n*°*24)*

*Because the older one was 16, and the second one was 9. It was the older one who managed the younger one, gave food, etc. There were reports of multiple absenteeism at school, from school, for this mother, but nothing happened. (Coordination nurse, female, 40–59 years old, n*°*29)*

In these families, some parents did not see the benefit of their child participating in a sporting activity, so their motivation to enroll them seemed low, although perhaps they were taken up by other priorities. For others, the financial aspect was underlined to explain why children had no engagement in sport or cultural activities. However, other children found in sport an escape from academic failure. But, faced with the child's behavioral difficulties, some parents abandoned any idea of integrating into a sports association. Other parents left their children to engage with screens, although doctors recommended that they did not do so:

*He has the phone. It's like medicine. It's very hard to calm him down. Since my girlfriend died, the way to calm his tears was the phone. We didn't show him the phone, he's four years old. […] I even took out a subscription for him. I did that because he was always taking my phone. I bought a good phone for him and took out a subscription. (Birth father*, ≥*60 years old, six children of whom one with PAE, n*°*19)*

This type of kinship is generally described, even outside the context of disability, as emanating from parents who have resigned, which is a marker for child welfare services of family dysfunction. But this distancing from children's neurodevelopmental problems can reveal family strategies to resist this form of disqualification, in the face of the child's disorders and his “non-conformity” or his distance from the dreamed child. It is also a resistance to the disqualification that these children can lead, particularly for foster or adoptive families, who are deemed unable to take care of them.

#### 3.3.3 Distance as protection or way to manage respite

For family assistants, distancing themselves from the child's difficulties could be a protection to avoid their skills being called into question or having the situation interpreted as their inability to meet the needs of the child or to set rules, which would likely result in the end of fostering.

*And all the more because I tell myself that now, when I see colleagues or others, at meetings, I talk about FASD and every time, I'm told, “well, a good framework”. Finally, it's a disease that child welfare does not know. And I say to myself, it's too bad because there are children who are reoriented, because they are difficult or whatever. Perhaps if we helped them a little more, maybe there would be fewer reorientations*. (Foster and adoptive mother, 40–59 years old, two biological, two fostered children and one adopted with PAE, n°66)

This foster mother focused on taking in care following a colleague and the way she perceived the child's character:

*So, the former referent said no to me, he is capricious. Yes. He is capricious, he has a hard head, he has this, he has that. And then, lots of things. I didn't want to keep him anymore because he puts the souk at home, he breaks everything, he throws everything away. Because I said, I had three children. I don't compare them to my children, be careful. But I saw that there was a problem*. (Foster mother, < 40 years old, three biological children, two fostered with PAE, n°33)

There were also foster families who respected the regulation of non-attachment to the child and who would give up on care when it went wrong, even if it was always violent for them as well as for the child. This was particularly likely to occur when their training was insufficient, notably if they never had heard of FASD:

*So, you can imagine how violent it is. It's doubly violent, actually. And also, how guilty it is for the family assistant. “Ah, but maybe I shouldn't have stopped”. And what she said was terrible, in fact, because this situation […] “Maybe if I had had this training before”, so all the work that we did there, there would not have been a break*. (Specialized educator, female, 40–59 years old, n°56)

Added to the distance taken by parents to meet the needs of the children was a sort of resignation from some professionals when facing families where the problems accumulated.

*The doctor has very immediate problems, very immediate concerns with this family there. And then my goodness they think they are all like that in the family, the mother also, older children, the stepfather: we know them. The social services of the city know them for 30 years. It's okay! I'm speechless!* (Network director, male, 40–59 years old, n°75)

In order to protect the family unit and not sacrifice other children, some families chose to remove the child with FASD by, for example, sending them to a boarding school, to limit the exposure to their difficulties, notably their behavioral disorders. Like this couple, who received this proposal from the Child Welfare service:

*Researcher: So you have him 24 hours a day with you?*

*Father: No, now no, it was too difficult to have him like that, he has been in an internship for a year and a few months*

*Researcher: Okay, how old is he?*

*Father: He is eight years old*

*(Trustee couple*,<*40 years old, two biological children and one foster child with PAE, n*°*76)*

Not getting too involved or asking for shared custody can also be a way to gain some respite, which is not easily organized for these families, especially for foster families, as this mother explains to us:

*Currently, yes, I am worried, because there are moments when (breath), I am a little tired! There you go and sometimes I ask for a little respite because, well just to be able to breathe a bit, because she's not going on a visit to her mom's and so it's one, there are moments it's a bit heavy anyway […] then there will be a relief that will be done an adapted structure there you go, which take the children for a weekend. I don't ask for every weekend, no, but at least one small weekend per month (Foster mother, less than 40 years old, 3 children of whom 1 PAE, n*°*2)*.

This shared parenting is increasingly observed in judicial or administrative decisions, on the model of that of divorced parents, to reorganize the care of children between both parents, between the foster family and the biological family, even between two foster families. Several examples have been reported to us, showing that in the face of significant difficulties for children, child protection services can also try to protect the health of foster mothers by reducing the time the children spend at their home. This is also the case when children are placed part-time in institutions and with their parents for weekends for example.

## 4 Discussion

We have chosen to cover parenthood in the broad sense by including foster families, who could also be considered professional caregivers, but who must behave like mothers (or fathers) without parental positions being named (Neyrand, 2005). Its definition is uncertain, and the dividing lines are blurred, especially because some have an adoption project or have adopted at least one of the children. The borders are especially porous as the child approaches adulthood, or retirement age for the foster families. We wanted to show that in this group of families too, parenthood could take various forms (Blythe et al., 2013). The types of styles drawn here have no doubt emerged because of the diversity of stories collected.

For parents, the FASD disability gives rise to an intense feeling of guilt (Korff-Sausse, 2007) which can add to the shame of not having borne the child for adoptive mothers or of having exposed them to alcohol for biological mothers (Zabotka et al., 2017). Even more than for children without disabilities, parenting involves a succession of experiences or challenges that are more or less difficult to face (Martuccelli, 2015). We resume the typology that emerged from our analysis to provide focus to this discussion.

### 4.1 Intensive parenthood

Intensive families showed their total involvement in all aspects of their children's lives by seeking the best care or unconventional alternatives, testing all educational methods and involving the children in numerous cultural or social activities, or sports. Mothers in particular appeared to be disproportionally involved to the point of giving up their professional career or considerably reducing it and developing the care of their children as a profession in its own right. The cost of their actions may be particularly high, especially if they forgo a salary, which could possibly cause financial difficulties for the whole family. Intensive parenting is now prevalent in many countries, linked with mothering and class privilege and constitutes the frame of the idealized parent, characterized as children-centered, expert-guided, emotionally absorbing, labor intensive and financially expensive. This type closely resembles what is described by Petrenko et al. (2019): in protective families, many actions are taken to counter the fate of the child forged by his disability, which in turn involves stressors.

The intensive family is the type most described in the literature, regardless of the disability, especially because the families interviewed are most often those who have the best resources, intellectual and financial, who want to testify and are involved in parents' associations. Regarding adoptive families, some have had a difficult infertility journey before the adoption project and therefore a special involvement in the child, including when he or she is accepted with specific needs (Toutain and Dartiguenave, 2015). These women mostly put the cause of their children before their own in order to be a model parent (García et al., 2018). These parents are referred to as “helicopter parents” by some authors, which is a parental style marked by constant supervision and excessive involvement, especially of mothers, in their child's life (Lee and Macvarish, 2020).

Once state schools fail, intensive families, particularly those with high cultural and economic capital, tend to enroll their children in private schools with alternative pedagogies like Montessori, Freinet or Steiner (Landour, 2015). This choice to move away from the traditional school for alternative modes of education leads some authors to name these mothers “mère première de cordée” (Proboeuf, 2021). Schools with a more flexible and less normative educational framework tend to be more aligned with their educational values, which emphasize autonomy, creativity and the development of the child rather than academic performance. Furthermore, they believe that these schools are more attentive to their requirements and sympathetic to the difficulties they encounter. These practices demonstrate a strong mobilization around the child, which is not just linked to a thorough monitoring of school standards but also to parental involvement (Landour, 2015).

Sports and cultural activities are not only considered crucial for these children's health but also as offering “learning opportunities” and success (Kleinert et al., 2007). The values conveyed by sport and music are considered a factor of resilience and these talents provide another model of success for these parents promoting self-esteem. These activities allow distancing of the stigmas of disability (Compte, 2010). The parents' excessive investment in the child may cause a rivalry between the parents and the child's medical or educational teams. As reported by Tessaire (2007) through an example, professionals “*feel that the parents, through all these steps, show that they have not accepted their daughter's disability and that they are trying to repair their disabled daughter*”.

This intensive parenting is, finally, also described in the literature as a source of stress, poor mental health and even suicidal thoughts, especially because the neuro-disabilities must be confronted throughout life and they realize that their involvement will be endless. This also implies that their child will never be autonomous, which leads them to implement guardianship measures to consolidate their future, although we do not have room to develop this theme here. The descriptions in the literature of adoptive parents of children with FASD show experiences summarized as “*different, difficult, and exhausting*” (Balcaen et al., 2021).

### 4.2 Inclusive parenthood

The second group of parents sought inclusion of their children with FASD disabilities in all domains of their lives, stressing all their efforts and fulfilments and looking for their happiness. They were more inclined to see the good side of things, the joyful or dreamy character of the children rather than their academic level. Their search for standardization led them to prefer public medical services, state schools in unmarked classes (often without specific help so as not to designate them as different) and team sports funded by the school or municipalities.

These parents sought to become the parents of children who are not defined by their disabilities by seeking their development without a primary focus on the disability (Korff-Sausse, 2007). The risk could be to ask too much of their child and go beyond their possibilities. Inclusive practice thus appeared to be a double process: disabled people must be accepted and integrated as they are, but they must integrate into the ordinary environment. This refers back to the question of “living together,” which implies reciprocity (Lacaze, 2014). These parents are doing everything in their power to keep their children from failing academically and socially with the aim of resembling other parents and making their children appear like other students. Their objective tends to be to maintain appearances to overcome the anxiety of being seen as incompetent parents (Swart et al., 2014).

The opinions expressed by parents in the general population are positive concerning inclusion for children with physical disabilities but less so for those with mental disabilities or behavioral disorders (Paseka and Schwab, 2020). These results explain why the effort made to include a child with FASD in an ordinary class is often very important for inclusive families, who generally must fight against the prejudices of other parents, potential rejection and strong demands from schools and care units. Resources in educational assistance staff are often limited, which makes this integration even more ineffective. The availability of information on FASD in sports clubs and the training of educators to care for children with FASD in collective structures are severely lacking, but remain essential for a successful integration. The stigmatization of sports practice for children or adolescents with these neuro-disabilities is exacerbated by the absence of training, which also goes against the initial objective of parents to alleviate stigma by fostering inclusion.

### 4.3 Distanced parenthood

In the third group, the notion of negligent parenting (Maccoby and Martin, 1983) did not seem completely in line with the statements made by our interviewees, so we prefer to talk about parenting with low demands. For some, these parents—especially in the descriptions of the professionals—seem to be resigned. They have given up putting the children in school to avoid having permanent negative feedback, even if they are forced to accept pedagogical contracts where the school required them, so the children only attend school one or two mornings per week. Perhaps due to a lack of knowledge, these parents do not understand the irreversible aspect of the neuro disabilities of FASD and end up accepting care late in an attempt to repair the children, get them back on their feet or alleviate school problems.

Biological parents who distance themselves from the problems of their children, including those due to their own health problem or addiction, more often lose custody—sometimes for a very long time. They do not respect the standards imposed by social services, and separation solutions tend to be preferred in the interests of the children (Simmat-Durand, 2005). In these families, parenting can be delegated to older siblings, who often already suffer from the disproportionate place that disabled children take in the family (Tessaire, 2007), and some authors do not hesitate to talk about resigning parents, for instance in the context of adoption (Ramos et al., 2015).

Foster families in particular tend to deal with matters in this way out of fear of seeing their professional activity called into question. They fear that the child's non-compliance would make them look incompetent. Distant parenting, in their case—but also for other families—could be a form of protection against disorganized children who are causing the family group to suffer. In all types of families, distance can be established by the use or imposition of boarding for children to rebalance the family group and offer more structured schooling of the child. In one case only, was boarding requested by a teen who wished to benefit from a framework.

Intensive parenting, as an ideal, requires financial and intellectual resources: “parents are expected to acquire detailed knowledge of what the experts consider proper child development and then spend a good deal of time and money attempting to foster it” (Scavarda, 2024). These resources are often beyond the reach of certain categories of families (Mollborn and Billingsley, 2025; Valentine et al., 2019). The parents of disabled children may distance themselves because, over the years and through their experiences, they are excluded from a whole part of the social world on a daily basis and rejected from the group of parents (Korff-Sausse, 2007). This phenomenon of disaffiliation is accentuated in the case of children placed outside their biological family, whose parents struggle to meet the demands of child protection services while hoping for a possible return. The foster families' investment in children's education is often considered to be lower, particularly in terms of ambitions, as in all extracurricular activities for which a budget is rarely allocated. For the same reasons, the use of private or alternative schools is not observed in these households. Foster caregivers have a contradictory task: to create a family without forming connections or replacing the birth families, even when they are not present. Should things become really bad, foster families can stop fostering, but with the risk that their skills may be questioned, and the discontinuity of care this decision implies (Brown et al., 2007).

This category also included biological families, notably under the responsibility of the fathers when the couple has separated because the addiction or the psychiatric health of the mother does not allow her to retain custody of the child (or when the mother has died). It can also occur, however, when it is a trusted third party in the family who assumes the parental role, in a less privileged environment, with exclusive recourse to free care (i.e., hospital, CAMSP), public schooling and leisure at home.

We also tended to find parents with heavy drinking in this category, coming from families with alcoholism over several generations. These are parents who are distant from the education of their children, but who sometimes have a very strong bond with them. This may refer to the notion of “good enough parenting” (Valentine et al., 2019). As for adults with Down Syndrome, making families for parents who are themselves with FASD was described throughout our interviews, because we collected stories from professionals in which the mother of the child had herself been affected by alcohol *in utero*. These situations were obviously experienced as failures by the medical teams, or with fatalism by the social workers, which eventually prevented intensive care for the children.

This research has made it possible to collect the voices of families that are difficult to reach, less often questioned like biological families, with varied cultural origins, but also families of all social levels in two very contrasted regions of France and through a national self-support group. This undeniably constitutes a strength of this material. The limits are obviously to have had to go through hospital services, only one GP having agreed to put us in touch with his patients. Our attempts to gather testimonies via social networks, by questionnaire, were not very fruitful, because the theme remains a sensitive or stigmatized subject.

## 5 Conclusion

Becoming a parent of children with disabilities is a long process, particularly when the origin is attributed to stigmatized behavior for both mothers and children; it requires adequate diagnosis and management to avoid loss of opportunity and to jeopardize the family cohesion. Parental investment, which determines the family trajectories, is based on social, financial and cultural resources and the ability to cope with family carers. The experiences of brothers and sisters were poorly mentioned by the parents we met, except to note the massive separations of siblings in the context of placements or adoptions, including in the case of twins. These separations were a source of concern for parents, who feared that their children might blame them one day for this loss in their lives. Many parents showed strong concern that, after their death, the siblings of the FASD children would have the responsibility to take care of them.

The disproportionate burden on women must be addressed, also because mothers raise alone children, not recognized by fathers, in families affected by addiction, or because couples have separated. Fathers obtain custody of the child when the mother has died or has a problematic use of alcohol. So, we have had great difficulty in involving fathers, because few of them are taking care of these children. These mothers ‘experiences show that care is not sufficiently structured, except in one French region, and that this absence of public structures leaves them alone, including because the prevention of FASD is almost absent.

Different attitudes are possible toward disabilities caused by PAE, as shown in the typology presented here. Family attitudes were independent of their legal status but were strongly influenced by the social, financial and intellectual resources that these families could mobilize. Common features between families in each type emerged, regardless of where the stories were collected and the cultural characteristics of the interviewees, which is a great strength of this analysis. The intensive family represents an ideal type in many rich countries, but there is the risk of developing stress and mental health problems for parents—especially mothers—often isolated or out of work, but also for the children themselves, who are subject to strong academic, sports, success pressures and despite their neuro-disabilities.

FASD and other neuro-disabilities lead to the isolation of parents, are sources of stress and affect the long-term mental health of both parents and children, and they constitute ACEs which in turn will generate addictions and mental health problems. These stressors have manifested, surprisingly, in all types of families and almost regardless of the educational type or their way of coping. This obviously raises the question of access to diagnosis and integrated care pathways, which would allow breaking the chain of these negative events. The families spoke of a fight, both against the neuro disabilities, but also against the prejudices of professionals, insufficiently trained, against rejection by school structures or during integration into the labor market. The professionals on their side felt very helpless facing the multiple needs of these families and also expressed suffering in their daily work to take them in charge. Policies for the prevention of fetal alcohol exclusively based on an abstinence during pregnancy message show here their limits. The issues expressed by families and professionals show the complexity of this consumption during pregnancy, which concern both women who are not able to limit their consumption and poorly informed women who wrongly interpret their consumption as safe. The efforts of professionals must focus on the advice to be given to all women wishing a pregnancy regardless of their social characteristics or their geographical or cultural origin.

## Data Availability

The datasets presented in this article are not readily available because 5 years restriction. Requests to access the datasets should be directed to laurence.simmat-durand@u-paris.fr.

## Appendix

### Precisions on French organization for disabled children

The clinical features associated with FASD present children with multiple problems. In France, the CAMSP is a medico-social establishment responsible for the early management of disability issues among children aged 0–6 years with difficulties or delays in their development (Buziau and Lanco Dosen, 2024; Toutain et al., 2007). Another in town structure is the CMPP, a center for children with learning difficulties, language or behavioral disorders, offering child or family support, mainly psychological consultations. Failing this, children with FASD are taken care of by health professionals working in the private sector, which means parents have to manage the multi-disciplinary care pathway (which can include a speech therapist, psychomotor therapist, psychologist, among others) of their children (Leruste et al., 2024).

In the French schooling system, children with disabilities can benefit from an assistant in ordinary classes, named AESH, but with the condition of having a recognition as disabled people that implies a diagnostic and the validation from a local commission (MDPH). Alternatively, they can be enrolled in a special education course, in ordinary schools with specialized classes called ULIS or CLIS depending on the period. There are a multitude of structures according to age and disability but they are often restricted to children with low intelligence quotient.
